# Stearoyl-CoA desaturase-1 is vital for milk lipid synthesis: Deletion impairs mammary gland and neonatal development

**DOI:** 10.1016/j.jlr.2025.100941

**Published:** 2025-11-11

**Authors:** Mugagga Kalyesubula, Jysiane Cardot, Hailey Huff, Daniel Bergman, Kaitlyn O'Donoghue, Veronica Pegkou Christofi, Kaela Groppel, Lucas M. O'Neill, Lucas Lefers, Jacqueline Rose Miller, Ethan Anderson, Madelaine M. Becker, Dylan Cootway, Joshua Walter, Leriana Garcia Reis, Linda Beckett, Christina R. Ferreira, Catherine Mounier, Isabelle Plante, Theresa M. Casey, James M. Ntambi

**Affiliations:** 1 Department of Biochemistry, University of Wisconsin-Madison, Madison, WI, USA; 2 Institut National de la Recherche Scientifique (INRS), Centre Armand-Frappier Santé Biotechnologie, Laval, Québec, Canada; 3 Department of Animal Sciences, Purdue University, West Lafayette, IN, USA; 4 Bindley Bioscience Center, Purdue University, West Lafayette, IN, USA; 5 Laboratoire du métabolisme des lipides, CERMO-FC, Département des sciences biologiques, Université du Québec à Montréal, Montréal, Québec, Canada; 6 Department of Nutritional Sciences, University of Wisconsin-Madison, Madison, WI, USA

**Keywords:** SCD1, lactation, milk, glycerolipids, lipidomics, triglycerides, diglycerides, mammary gland

## Abstract

The mammary gland synthesizes and secretes nutrient-rich milk containing lactose, protein, and lipids, with the complex assortment of lipids providing more than half of the energy and bioactive factors that impact the growth and development of neonates. The birth of neonates initiates the lipogenic capacity of the mammary gland with upregulation in expression of lipogenic genes, including Stearoyl-CoA desaturase (*Scd1*). SCD1 plays a critical role in lipogenesis, catalyzing the conversion of saturated fatty acids to monounsaturated fatty acids. Previous studies of *Scd1* knockout mice revealed that SCD1 impacts several metabolic processes in the liver and adipose tissue, including fat synthesis. However, the role of SCD1 in lactation is not fully understood. Our study aimed to determine the role of SCD1 in lactation and the effects of maternal knockout of *Scd1* on the growth of the lactating neonates. We employed second-parity *Scd1*-deficient female mice (n = 7) that we compared with wild-type mice (n = 6). To determine lipid and metabolic alterations, mammary gland and milk samples were harvested on lactation day 10. Relative to wild-type mice, mammary gland weight, alveolar area, and milk glycerolipid content were reduced in lactating *Scd1-*deficient mice. *Scd1* deficiency also diminished mammary gland biosynthetic metabolic pathways, such as glycerolipid and phospholipid synthesis, while enhancing catabolic pathways, such as fatty acid oxidation. Neonates nursed by *Scd1-*deficient mice exhibited lower body weights. These findings highlight the critical role of SCD1 in orchestrating metabolic adaptations during lactation to ensure adequate milk synthesis to support the rapidly growing neonates.

## Graphical abstract

**Figure:**
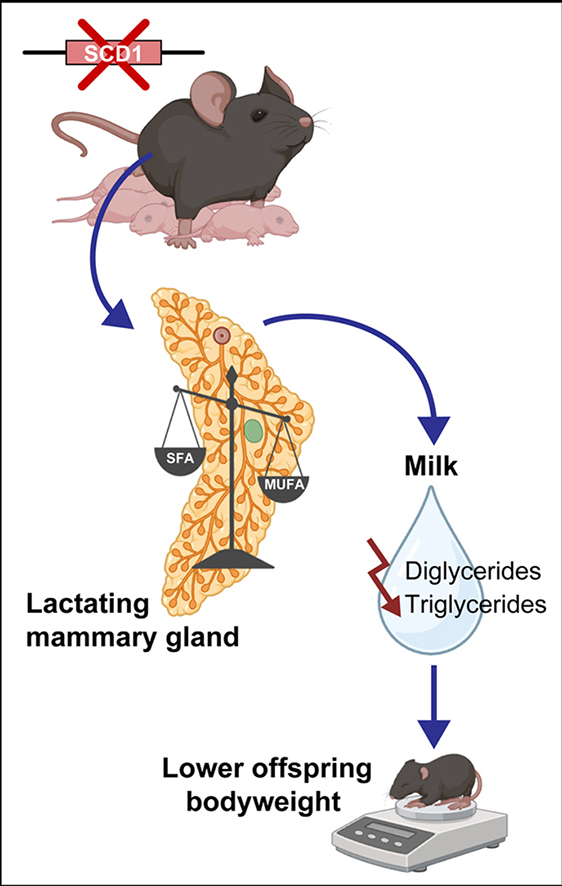

The mammary gland is crucial for mammalian development, secreting nutrients and bioactive factors such as milk for neonatal survival and growth during lactation. Post-natal mammary gland development is orchestrated by ovarian hormone-induced elongation and branching of the ductal system during puberty. The mammary gland reaches its functional stage through sequential phases of development and differentiation beginning at the onset of pregnancy (proliferation, secretory differentiation, secretory activation, and lactation), which are regulated by hormonal signals (1, 2). The proliferative phase, during early pregnancy, establishes fully expanded ducts and milk-producing lobulo-alveolar structures (2, 3). Secretory differentiation begins in mid-gestation and marks the triggering of biochemical changes reflected in the upregulation of key enzymes that regulate lipid, protein, and lactose biosynthesis, rendering the mammary gland pre-lactational by the end of pregnancy (1, 2, 3). Periparturient changes in the hormone milieu, including the drop in progesterone and rise in glucocorticoids and prolactin, initiate secretory activation, leading to the onset of milk production by the mammary gland's acini (1, 2). Lactation ensues, as does the continued milk production, in response to the secretion of prolactin and oxytocin caused by the suckling of neonates (1, 4).

Triglycerides, composed of a glycerol backbone and three fatty acyl groups, make up 98% of the lipids in milk fat (1, 5). The fatty acyl groups are either sourced from the maternal diet, adipose stores, or de novo synthesized by the mammary gland (5, 6). Mice fed a standard rodent chow diet of about 8% kilocalories from fat rely primarily on de novo synthesis of fatty acids using glucose and amino acids as precursors to provide adequate milk fat to their suckling neonates (1). Lipids in milk provide approximately 50% of the caloric needs of neonates (7). The mammary gland's capacity for de novo lipid synthesis commences shortly after parturition, being mediated by a dramatic increase in the expression of lipogenic enzymes, including *Fasn* and *Scd1* (8).

The mammary gland secretes fats via an apocrine process, beginning with the release of lipid droplets assembled in the rough endoplasmic reticulum of the cell (9). A monolayer of phospholipids surrounds the primarily triglyceride core of lipid droplets that coalesce and become larger as they move toward the apical surface of the mammary epithelial cell. Upon reaching the apical surface, lipid droplets associate with the cell's phospholipid bilayer membrane, which surrounds the droplet as the milk fat globule unit is extruded from the cell. The milk fat globule formed during the secretion process consists of a phospholipid trilayer surrounding a neutral lipid core. Mouse studies revealed that the milk fat globule membrane proteins Butyrophilin 1a1 (BTN1A1) and Xanthine oxidoreductase (XOR) play essential roles in milk fat secretion. Genetic ablation of Btn1a1 (*Btn1a1(−/−)*) resulted in the accumulation of fats within the cytoplasm, abnormal milk fat secretion, and high rates of pup death (10). Pups of mice with a heterozygous knockout of XOR (*Xor+/−*) also died of starvation prior to weaning. Histological analysis of mammary glands of *Xor* ± mice showed signs of premature involution, whereas analysis of electron microscopy images supported a role for XOR in mediating the enveloping of milk fat droplets with the apical plasma membrane prior to secretion (11).

SCD1 catalyzes the conversion of saturated fatty acids (SFA) to monounsaturated fatty acids (MUFA), primarily converting palmitate (C16:0) to palmitoleate (C16:1) and stearate (C18:0) to oleate (C18:1) (12, 13). These MUFA products serve as essential building blocks for the synthesis of various complex lipids, such as diglycerides (DG), triglycerides (TG), phospholipids, and cholesterol esters (13, 14). Studies of genetically modified mice found that *Scd1* deficiency significantly impacted lipid metabolism in the liver, a key lipogenic tissue, leading to reduced body fat accumulation, a decrease in monounsaturated lipid levels, and consequently lower body weights (12, 15).

Generation of *Scd1* knockout models enabled elucidation of the role of SCD1 in liver and adipose lipogenesis (12, 16, 17). Whole-body knockout and liver-specific knockout mice demonstrated that *Scd1* deficiency decreases hepatic de novo lipogenesis (12). However, the role of SCD1 in the mammary gland, an organ with uniquely high lipid synthetic activity during lactation, is not fully understood. We, therefore, investigated the effect of *Scd1* deficiency on the mammary gland and milk lipid profile during lactation and its subsequent effect on neonatal weight. We hypothesized that *Scd1* is essential for sustaining the lipid biosynthetic capacity of the lactating mammary gland, and that its absence would alter milk lipid composition and impair neonatal growth. Our findings showed that *Scd1* deficiency diminished the mammary gland's ability to synthesize milk glycerolipids, reducing neonatal weight, and thus underscoring the key role of SCD1 in lactation.

## Materials and methods

### Animal study design

All studies involving animals were reviewed and approved by the Institutional Animal Care and Use Committee guidelines of the University of Wisconsin-Madison (protocol # A005125) prior to beginning experiments. The mice in this study were in the C57BL/6 genetic background and were fed a standard rodent chow diet (Purina 5008; Harlan Teklad) with ∼6.5% fat (18) until being changed to a mouse breeder diet (Purina 5015 with ∼11% fat (19) when paired with a male. The process of generating *Scd1*−/− (*Scd1* global knockout (GKO)) has been previously reported (12).

In this study, we utilized wild-type (WT; n = 6) and GKO (n = 7) female mice and bred them twice. All dams were generated by mating heterozygous females with heterozygous males. Mice were initially paired at ∼7 weeks of age, permitting a complete reproductive cycle and allowing dams to nurse their pups. After weaning, mice were re-paired to generate a second litter. Thus, all studies were conducted on second parity dams, so all growth data for neonates, milk, and mammary glands are from second parity dams. To ensure that all pups were of the same genotype, GKO females were bred with WT males, while WT females were bred with GKO males, resulting in heterozygous offspring. Pregnancy was closely monitored, and litters were standardized to 5 pups per dam on postnatal day 2, with surplus pups cross-fostered to other mice within the colony.

### Mouse milk and tissue collection

**Fig. 1:**
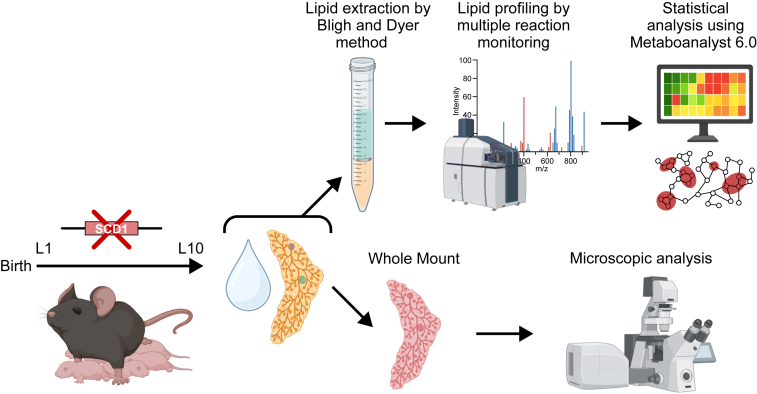
Schematic of the research study design: Milk and mammary glands were collected from wild-type and *Scd1-*deficient (GKO) mice on day 10 of lactation (L10), followed by lipid and metabolite extraction using MRM-profiling analysis and whole mount analysis. Designed with Biorender.

Milk samples were collected from dams on postnatal day 10, utilizing a previously established protocol (20). Briefly, following a 2-h separation from their pups, dams were anesthetized with isoflurane and administered 0.2 ml of oxytocin (20 USP/ml) IP to stimulate milk letdown. Approximately 400 μl of milk was collected using an electric breast pump (Swing Breast Pump, Medela) (20). The milk was immediately snap-frozen in liquid nitrogen and stored at −80°C until further analysis. After milk collection, dams were euthanized via isoflurane overdose, followed by the collection of fourth inguinal mammary glands for downstream analysis (Fig. 1).

### Lipidomics

Lipid and metabolite extraction was performed according to the Bligh & Dyer protocol (21). Milk samples were prepared for extraction by mixing 50 μl of milk with 150 μl deionized water (DI H_2_O). For mammary gland extracts, 20 mg of tissue was homogenized in 150 μl of DI H_2_O with 1.4 mm ceramic (zirconium oxide) beads using the Precellys24 tissue homogenizer (Bertin Technologies), and then brought to a final volume of 200 μl using DI H_2_O for extraction.

To run the discovery phase of MRM (Multiple Reaction Monitoring)-profiling, pooled milk samples, and pooled mammary gland samples were created by taking aliquots from individual extracts from all samples to generate four pooled samples by genotype and tissue or milk. All tubes were dried in a centrifugal evaporator (Savant SpeedVac AES2010, ThermoFisher Scientific). Dried lipid extracts were stored at −80°C until mass spectrometry analysis.

MRM profiling analysis was conducted in two phases: discovery and screening. For the discovery phase, samples pooled by genotype and sample-matrix (4 pools) were profiled for a list of MRMs related to lipid species generated by combining the *m/z* for the molecular ion based on the LIPID MAPS® online database and a class- or fatty acyl loss-specific product ion (22, 23). Of the MRM profiled in the discovery phase, those found to be at least three-fold higher than the blank in at least one of the pooled samples were then used to interrogate individual samples in the screening phase of the MRM profiling analysis (23). Raw data files were uploaded to PURR (DOI: 10.4231/JA2N-DJ86).

Due to the limited time of signal provided by the sample at flow injection, the MRMs profiled in the screening phase were organized into 7 methods (24), each one involving 2 min of data acquisition for a maximum of 445 MRM transitions. Quality control (QC) samples were interspersed throughout the run. Dried milk sample extracts were reconstituted in 550 μl of injection solvent consisted of acetonitrile (ACN) + methanol (MeOH) + 300 mM ammonium acetate (NH4Ac) 3:6.65:0.35 (v/v/v) + 60 μl of chloroform (CHCl3) with 0.01% butylated hydroxytoluene (BHT), and mammary gland extracts were resuspended in 350 μl of injection solvent + 20 μl of CHCl3 with 0.01% BHT, composing the stock solution. The solvent used to dilute lipid extracts for injection was spiked with 0.05 ng/μl of EquiSPLASH LIPIDOMIX isotopically labeled lipids (cat. no. 330731, Avanti Polar Lipids).

For metabolite evaluation, 400 μl of ACN: DI H2O (50:50) was used to resuspend the metabolites for both samples.

The reconstituted extracts were individually diluted 500X for milk and 300X for mammary gland tissue with injection solvent ACN + MeOH + 300 mM NH4Ac 3:6.65:0.35 (v/v/v), containing internal standard (IS) at 0.05 ng/μl. The screening phase of the MRM profiling was conducted by injecting 8 μl of the diluted lipid extract into the ESI source of an Agilent 6410 QQQ mass spectrometer (Agilent Technologies) using a micro-autosampler (G1377A). The precursor ion selection window was 0.7 Th. The capillary pump connected to the autosampler operated with a flow of 10 μl/min and a pressure of 150 bar. The capillary voltage on the instrument was 3.5–5 kV, and the gas flow was 5.1 L/min at 300°C.

The ion intensities of all MRMs were recovered, and each lipid ion was normalized by the ion intensity of the corresponding isotopically labeled internal standard (IS) (normalization by IS) within each lipid class (phosphatidylcholines (PC), phosphatidylethanolamines (PE), phosphatidylserines (PS), phosphatidylinositol (PI), phosphatidylglycerol (PG), diglycerides (DG), triglycerides (TG), ceramides (Cer), sphingomyelins (SM), cholesteryl ester (CE) to acquire semi-quantitative amounts. Subsequently, all data were consolidated into a unified file for each matrix (milk and mammary gland).

### Whole mount

The inguinal mammary glands were harvested and stretched using forceps to regain their original shape on a microscope slide. To fix the tissue, the slides were immersed in a bath of Carnoy's fixative (100% EtOH, chloroform, glacial acetic acid) for 4 h at room temperature. They were gradually rehydrated in alcohol baths (95%, 75%, 50%, and 25% EtOH). The slides were stained in carmine alum (2% carmine and 5% potassium aluminum sulfate dissolved in sterile H_2_O) for 3 h to stain the mammary epithelium. The tissues were then gradually dehydrated in alcohol baths (25%, 50%, 75%, and 95% EtOH) and incubated overnight in xylene to clear fat (25). The stained whole mounts were imaged at three different places around the edge of the mammary gland using a Nikon AR1 confocal at 10x magnification. The area of the acini was measured using ImageJ analysis software.

### RNA isolation and real-time quantitative PCR

Mammary gland and liver RNA was extracted using Trizol reagent (Life Technologies/Invitrogen, Carlsbad, CA, USA), following homogenization of tissues with a TissueLyzer II (Qiagen), and subsequently treated with TURBO DNase (Ambion) to eliminate genomic DNA. Total RNA was evaluated for quantity and quality using the uDrop plate and Varioskan LUX (Thermo Fisher Scientific). cDNA was synthesized from 1 μg of total RNA using the High-Capacity cDNA Reverse Transcription Kit (Applied Biosystems). Real-time qPCR was performed with the PowerUp SYBR Green 2x Master Mix (Thermo Fisher Scientific) on an Applied Biosystems QuantStudio 5 Real-Time PCR System in a 384-well plate. Relative expression levels of target genes were quantified by the ΔΔCT method (26), with normalization to the expression of *Rps3*, serving as a reliable housekeeping gene. Details of the primer sequences can be found in Supplemental Table 1.

### Data and statistical analysis

Metaboanalyst 6.0 (27) was employed for data visualization and statistical analysis of MRM data. The analyzed lipids were normalized using internal standards. Following upload, the data were normalized by auto-scaling and checked for normal distribution. Multivariate statistical tests, including principal component analysis (PCA) and hierarchical cluster analysis, and univariate tests, including the *t* test, were used to identify lipids and metabolites significantly influenced by genotype. Lipid species were considered significant if they had a ≥1.2-fold change and an FDR-corrected *P*-value of 0.05. The Benjamin-Hochberg procedure for FDR correction was employed. Due to the absence of significant metabolites following FDR correction, a raw *P*-value cut-off of 0.05 was employed to identify differentially distributed metabolites. To gain further insight into changes in lipid profiles due to loss of *Scd1*, the lipid species that differed between WT and GKO mice in TG, DG, and PC classes were categorized according to total carbon length and number of double bonds, and then graphed as a percentage of the total number elevated or reduced in the GKO strain versus WT. The pup weight was analyzed with nested ANOVA with dam as a nested factor and dam weight as a covariate. Statistical analysis for the rest of the data was performed in JMP using a Student's *t* test. Significance was considered when *P <* 0.05. Data are presented as mean ± SEM. GraphPad Prism was employed for data visualization.

## Results

### *Scd1* deficiency decreases mammary gland and neonatal weight

**Fig. 2:**
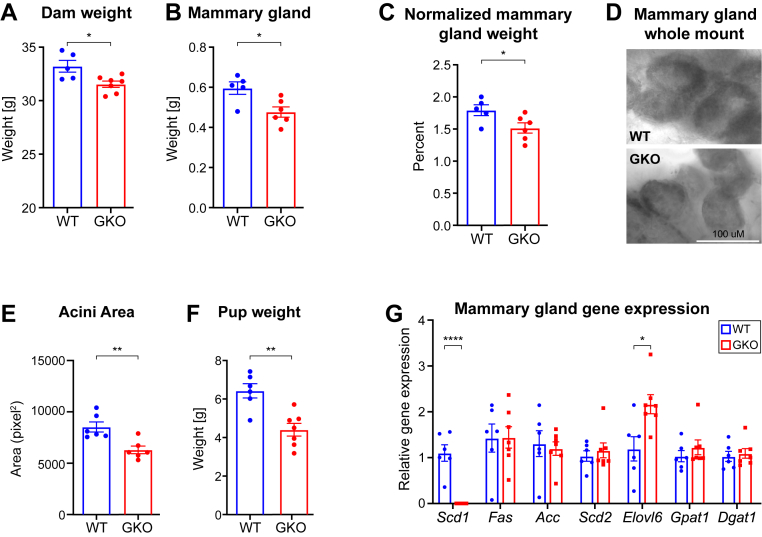
*Scd1* deficiency decreases mammary gland weight and acini area, and pup weight by lactation day 10. A: Dam weight, B: inguinal mammary gland weight, C: and relative mammary gland weight (percentage of the body weight). D: Confocal images of the mouse mammary gland whole mounts showing the acini and E: average acini area by genotype. F: Average pup weight on lactation d 10, with dam as a nesting factor and dam weight as a covariate. G: Relative lipogenic gene expression of the mammary gland ∗*P* < 0.05, ∗∗*P* < 0.01, ∗∗∗*P* < 0.001, ∗∗∗∗*P <* 0.0001. Data are presented as Mean ± SE. GKO: *Scd1* global knockout mice. WT: Wild-type control mice.

To determine if SCD1 plays a role in mammary gland development and milk production, we utilized lactating mice with global *Scd1* ablation (GKO). The *Scd1*-deficient dams, which exhibited lower body weights (Fig. 2A), also displayed reduced inguinal mammary gland weight during lactation (Fig. 2B). Even after adjusting for the dams' body weight, GKO dams maintained lower mammary gland mass (Fig. 2C), suggesting that *Scd1* deficiency intrinsically leads to decreased weight of mammary glands during lactation. Given that the mammary glands of GKO mice were lighter than their WT counterparts, we assessed the mammary gland architecture. Whole-mount analysis of the mammary glands revealed a decrease in the size of the acini (Fig. 2D, E). Moreover, pups nursed by *Scd1* deficient dams, despite having the same genotype as those nursed by wild-type dams, exhibited lower body weights after 10 days of lactation (Fig. 2G). As previously observed in gonadal white adipose tissue (28), mammary gland lipogenic gene expression was largely unaffected, except for elevated *Elovl6,* which codes an enzyme that catalyzes the chain elongation of C12-16 fatty acids to C18 fatty acids (29, 30) (Fig. 2F). Together, these findings indicate that SCD1 ablation alters acini development and milk biosynthesis, impacting neonatal growth during lactation.

### *Scd1* deficiency alters the mammary gland lipidome, decreasing glycerolipids and phosphatidylcholines

The finding of impaired pup growth led us to probe the lipid landscape of the mammary gland and milk of *Scd1*-deficient mice. The impact of genotype on the concentration of each lipid class was analyzed by summing individual lipids after normalizing their concentration to the internal standards (Table 1). In the mammary gland, the concentration of phosphatidylcholines (PC) tended to be lower (*P =* 0.12) in the GKO mice. In the milk, the concentration of DG was significantly reduced (*P* = 0.03) in GKO mice, and TG levels tended (*P* = 0.07) to be lower in *Scd1* knockout versus WT mice (Table 1).

**Table 1:** Impact of genotype on lipid class concentration in the mammary gland and milk of wildtype (WT) and global *Scd1* knockout (GKO) mice

Lipid Class	WT	GKO	*P* Value
Mammary Gland			
Cholesterol ester (μg/ml)	12.80 ± 1.90	11.67 ± 0.90	0.60
Phosphatidylcholine (μg/ml)	9,566 ± 997.7	8,119 ± 710.7	0.12
Phosphatidylethanolamine (μg/ml)	1,371.6 ± 446	1,388.8 ± 409.1	0.98
Phosphatidylinositol (μg/ml)	158.03 ± 41.5	167.03 ± 41.8	0.88
Phosphatidylserine (μg/ml)	141.12 ± 39.2	140.16 ± 38.4	0.99
Phosphatidylglycerol (μg/ml)	2,418.5 ± 724.5	1,774.6 ± 536.4	0.49
Triglycerides (μg/ml)	25,789.3 ± 3,240.8	26,504.9 ± 1815.6	0.85
Diglycerides (μg/ml)	4,850.8 ± 493.7	3,828.7 ± 613.54	0.22
PML/TG^a^	0.56 ± 0.06	0.46 ± 0.08	0.39
Milk			
Cholesterol ester (μg/ml)	8.85 ± 1.05	12.05 ± 2.6	0.30
Phosphatidylcholine (μg/ml)	1,161 ± 182.5	1,160 ± 123	0.99
Phosphatidylethanolamine (μg/ml)	67.9 ± 29.8	71.3 ± 23.6	0.93
Phosphatidylinositol (μg/ml)	8.46 ± 3.3	8.58 ± 2.0	0.98
Phosphatidylserine (μg/ml)	129.7 ± 36.7	116.9 ± 47.1	0.83
Phosphatidylglycerol (μg/ml)	3,077.7 ± 1,041.2	2,176.9 ± 862	0.52
Triglycerides (μg/ml)	35,268.2 ± 1918.9	19,569.3 ± 7,022.6	0.07
Diglycerides (μg/ml)	11,454.7 ± 869.6	5385.8 ± 2019.1	**0.03**
PML/TG^a^	0.12 ± 0.03	0.29 ± 0.09	0.12

a Ratio between membrane lipids (PML) and triglycerides (TG). Statistically significant *P*-values are shown in bold.

**Fig. 3:**
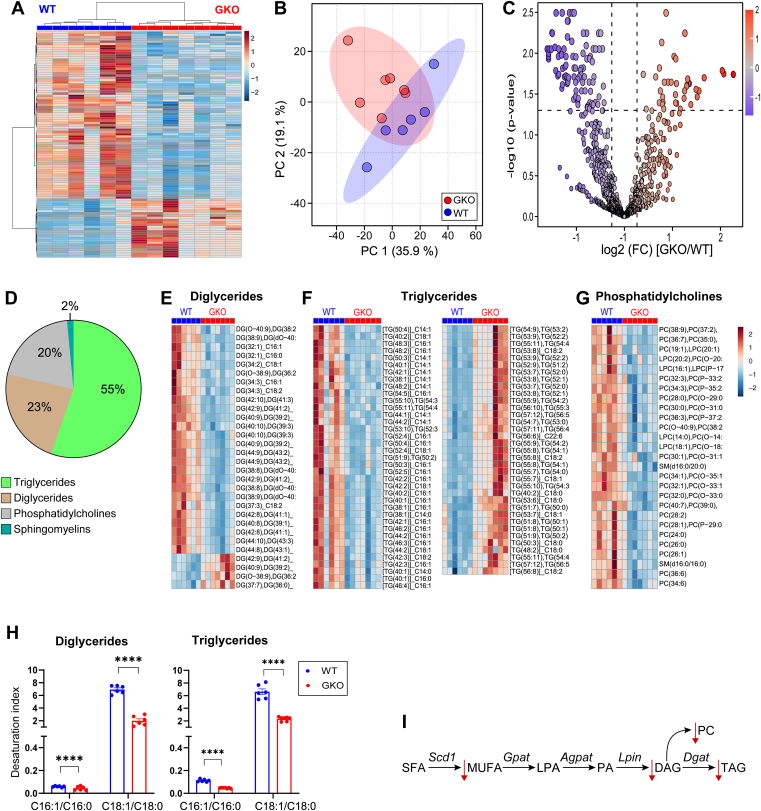
*Scd1* deficiency reshapes mammary gland lipidome during lactation, decreasing glycerolipids and phosphatidylcholines. A: Heatmap and dendrogram of hierarchical cluster analysis of the top 100 differentially abundant lipids by *P*-value in the mammary gland between WT and GKO mice. B: Scores plot of principal component analysis of mammary gland lipid profiles of WT and GKO mice. C: Volcano plot illustrating differential lipids in the mammary glands of WT and GKO mice. Lipids that are increased by 1.2-fold in GKO/WT mice with a *P* value of 0.05 after FDR correction are on the right. Those decreased are on the left. D: Distribution of the lipids differentially abundant in the mammary gland by class. Heat map of differentially abundant lipids in E: DG, F: TG (TG decreased in GKO are shown on the left; those elevated in GKO are shown on the right), and G: PC classes. H: The desaturation index of triglycerides and diglycerides. I: Schematic proposing that the absence of *Scd1* led to lower MUFA conversion from SFA (from diet or de novo synthesized), ultimately lowering DG, PC, and TG. Lipid identifiers include the total number of carbons and double bonds (e.g., TG(53:7)). For TG and DG species, an additional annotation (e.g., _C18:0) specifies one of the individual fatty acid chains. GKO: *Scd1* global knockout mice. WT: Wild-type control mice. DG: Diglycerides, TG: Triglycerides, PC: Phosphatidylcholines.

Further analysis of the principal component and hierarchical clustering of mammary gland lipid profiles demonstrated clear genotype-dependent segregation of samples (Fig. 3A, B). *t* test, with multiple corrections, identified 130 significantly (FDR<0.05) altered lipids out of 886 MRM profiles, with 91 lower and 39 greater in mammary glands of GKO than WT mice (Fig. 3C, Supplemental Table 2). Notably, TG, DG, and PC accounted for the majority of altered lipids (55%, 23%, and 20%, respectively) (Fig. 3). Strikingly, 87% of DG and 51% of TG were decreased in *Scd1*-deficient mammary glands, while all PCs were diminished (Fig. 3E–G). These findings reveal that SCD1 affects the lipid profile of the mammary gland during lactation.

Given SCD1's catalytic role in converting saturated fatty acids (SFAs) to monounsaturated fatty acids (MUFAs), specifically palmitate (C16:0) and stearate (C18:0) to palmitoleate (C16:1) and oleate (C18:1), respectively, prompted an examination of the desaturation index of TG and DG. Our results show that *Scd1* deficiency results in a significant decrease in the desaturation indices of both TG and DG (Fig. 3H). These results highlight SCD1's essential function in converting SFAs to MUFAs for incorporation into various lipid species. The absence of SCD1 likely hinders this conversion, leading to reduced MUFA availability, which in turn constrains the synthesis of DG, PC, and TG (Fig. 3I). Taken together, our findings suggest that *Scd1* deficiency reduces mammary MUFA synthesis and lowers the synthesis of key lipid species such as DG, TG, and PC.

### *Scd1* deficiency affects the carbon length and unsaturation profiles of the mammary gland lipids

**Fig. 4:**
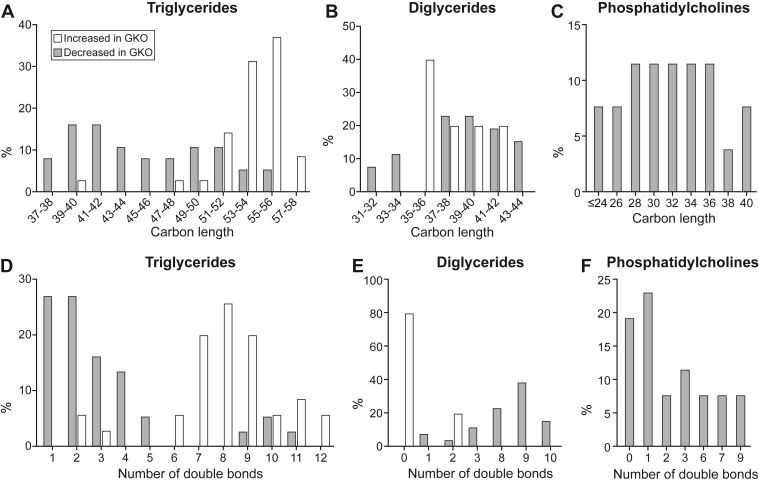
*Scd1* deficiency impacts the distribution of TG, DG, and PC classes of lipids in the mammary gland by carbon length and level of unsaturation. The percentage of lipids significantly increased or decreased in GKO mouse mammary glands were analyzed for their distribution by the number of total carbons across fatty acyl groups within A: triglycerides, B: diglycerides, and C: phosphatidylcholines class, as well as number of double bonds across the fatty acyl groups within D: triglycerides, E: diglycerides, and F: phosphatidylcholines in the mammary gland of GKO mice. Note: Significantly altered lipid changes in GKO mice predominantly showed reductions, with fewer or none exhibiting increases in certain lipid classes. The data is represented as a percentage of the total difference, categorized by carbon length or double bond. GKO: *Scd1* global knockout mice.

We subsequently examined the carbon length distribution of the significantly altered TG, DG, and PC. Notably, the TG greater in abundance in *Scd1* deficient mice had a total carbon length of 53–58 (Fig. 4A), indicating that fatty acyl groups of the TG in greater proportion were primarily sourced from circulation, as lengths were greater than 17 carbons (31). In contrast, the decreased TG spanned across an extensive range of carbon lengths, with 70% being ≤46 carbons (Fig. 3A), implying a lower rate of de novo synthesis in GKO mice (31). All DG greater in abundance in the GKO mammary gland had ≥35 carbons across the two fatty acyl chains, whereas those lesser in abundance ranged from 31 to 44 carbons (Fig. 4B). The PC lower in GKO ranged from 14 to 40 carbons (Fig. 4C), indicating a decrease in fatty acyl groups averaging 7–20 carbons in length, again pointing to a reduction in de novo lipid synthesis in GKO mice.

Analysis of the number of double bonds by lipid class was also conducted. The TG greater in GKO mammary glands displayed a notable enrichment of 8 double bonds across the 3 fatty acyl groups (Fig. 4D), reflecting a higher amount of dietary PUFA incorporation into milk fats. Conversely, the decreased TG exhibited a pronounced shift toward 1 and 2 double bonds (Fig. 4D), indicative of higher MUFA levels (Fig. 3H). The DG greater in GKO showed a prominent peak of full saturation across both fatty acyl groups, with nearly 80% bearing fully saturated acyl chains (Fig. 4E), reflecting an accumulation of saturated lipids. The decreased PC displayed a predominance of a single double bond (Fig. 4H), implying that a high proportion of MUFAs became less available for lipid synthesis upon *Scd1* deficiency.

### *Scd1* deficiency lowers milk lipids

**Fig. 5:**
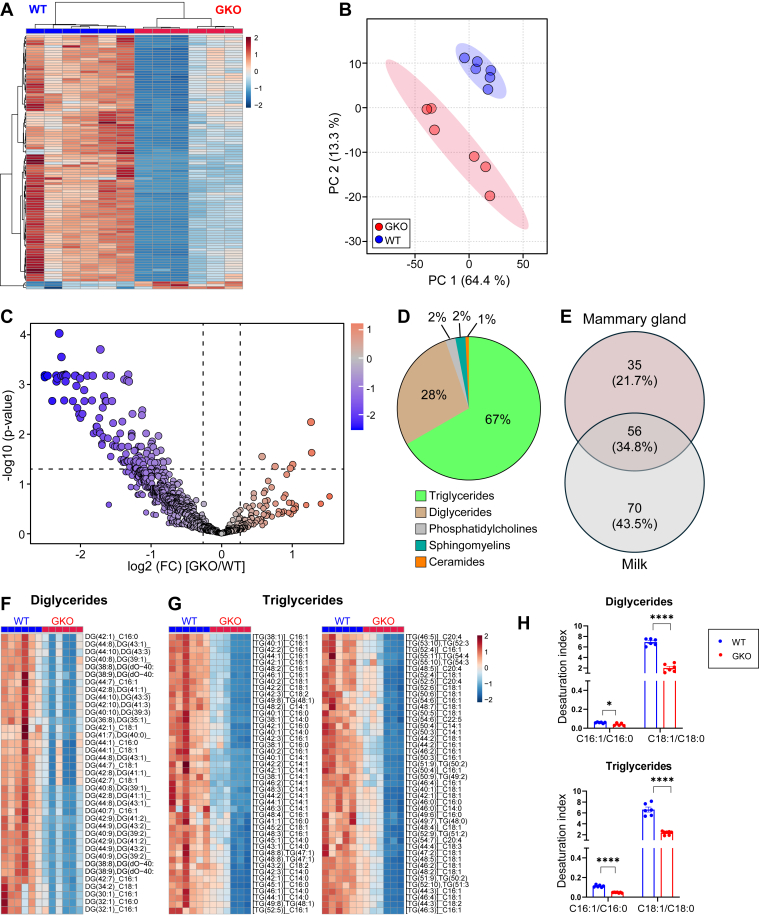
*Scd1* deficiency alters milk lipid profiles. A: Heatmap and dendrogram of hierarchical cluster analysis of the top 100 milk lipids by *P*-value of WT and GKO mice. B: Scores plot of principal component analysis of milk lipid profiles of WT and GKO mice. C: Volcano plot illustrating differential lipids in the milk of WT and GKO mice. Lipids that are increased by 1.2-fold in GKO/WT mice with a *P* value of 0.05 after FDR correction are on the right. Those decreased are on the left. D: Distribution of the lipid altered in milk by class, and E) Venn diagram of analysis of significantly decreased lipids overlapping and unique to the mammary gland and milk of WT and GKO mice. Heat map of differentially abundant lipids in F: DG and G: TG classes. H: The desaturation index of triglycerides and diglycerides. Lipid identifiers include the total number of carbons and double bonds (such as TG(53:7)). For TG and DG species, an additional annotation (such as _C18:0) specifies one of the individual fatty acid chains. GKO: *Scd1* global knockout mice. WT: Wild-type control mice. DG: Diglycerides. TG: Triglycerides.

Lipid profiles of milk samples showed a clear segregation according to genotype, in both PCA and hierarchical cluster analysis (Fig. 5A, B). Among the 791 MRM of lipid profiles, 132 were significantly altered by genotype, with a notable decrease of 126 lipids in GKO mice and an increase of just 6 (Fig. 5C, Supplemental Table 3).

Notably, among the significantly altered milk lipids, TG and DG species predominated, accounting for 67% and 28% of total changed lipids, respectively (Fig. 5D). Unlike the mammary gland, where PCs constituted ∼20% of the significantly altered lipid species, their representation in milk was markedly lower at ∼2%. Overlap between the lipids altered by genotype in the mammary gland and milk was found, with 61% of decreased lipids in the mammary gland also reduced in milk (Fig. 5E). Moreover, all significantly altered milk TG and DG exhibited decreased levels in the milk of GKO mice (Fig. 5F, G). Interestingly, although all milk TGs were reduced in GKO dams, approximately half exhibited a more pronounced decrease (Fig. 5G). In accordance with the lipidomic alterations observed in the mammary gland, GKO mouse milk also exhibited a pronounced reduction in the desaturation index of TG and DG (Fig. 5H). These findings show that SCD1 impacts milk glycerolipid composition and also that changes in mammary gland glycerolipids are reflected in the milk.

### *Scd1* deficiency affects the carbon length and unsaturation profiles of the milk lipids

**Fig. 6:**
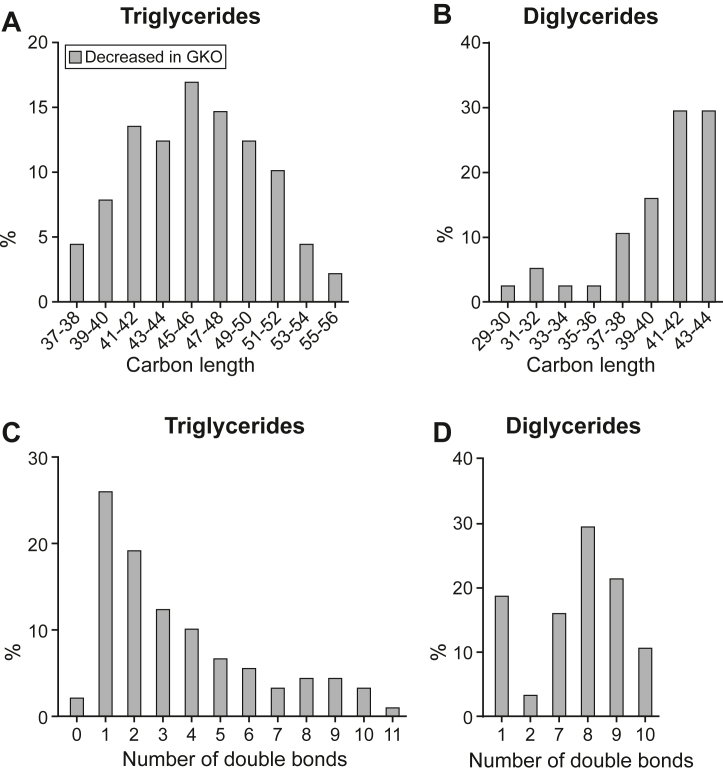
*Scd1* deficiency affects milk carbon length and level of unsaturation. The percentage abundance of total carbons within a lipid class significantly decreased in A: triglycerides and B: diglycerides in the milk of WT mice. The number of double bonds in significantly decreased C: triglycerides and D: diglycerides in the milk of GKO mice. Note: Most lipids, including all significantly altered triglycerides and diglycerides, were decreased in the milk of GKO mice; therefore, no distributions of total carbons and double bonds from increased species are depicted. GKO: *Scd1* global knockout mice.

After determining that milk lipids were impacted by *Scd1* deficiency, we then assessed the number of carbon chain lengths and unsaturation levels in the affected species. Reduced TGs in GKO milk spanned from 38 to 55 carbons (Fig. 6A), consistent with acyl group patterns characteristic of mammary-derived lipids. These TGs predominantly featured a single double bond (Fig. 6C), paralleling the mammary gland TG profile and underscoring SCD1's role in mammary gland MUFA synthesis. DGs also showed a similar trend: those diminished in GKO milk ranged between 30 and 44 carbons (Fig. 6B), closely resembling lipid species depleted in the mammary gland. Surprisingly, a majority (70%) of these decreased DGs were highly polyunsaturated, harboring seven or more double bonds (Fig. 6B). Collectively, our findings demonstrate that alterations in SCD1, a pivotal enzyme in lipogenesis, have a profound impact on mammary gland lipid metabolism during lactation, consequently influencing milk lipid composition.

### *Scd1* deficiency impacts mammary gland metabolites

**Fig. 7:**
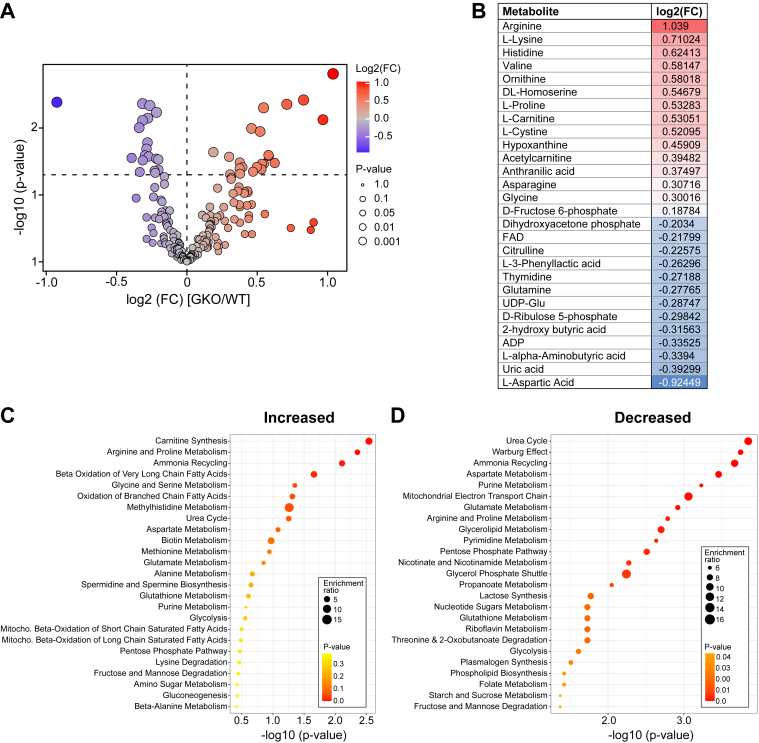
*Scd1* deficiency alters mammary gland metabolites. Mammary gland metabolomic analysis. A: Volcano plot of increased and decreased metabolites. Metabolites that are increased in GKO mice with a *P*-value of 0.05 are labeled in red. Those decreased are labeled in blue. B: Significantly altered metabolites. Color-code shading denotes the level of alteration, with the most increased in GKO mice shaded deepest red and the most decreased deepest blue. Small Molecule Pathway Database (SMPDB) pathway analysis of C: increased and D: decreased enriched metabolite sets.

Building on the lipidomic landscape, we performed metabolomic analysis to determine the metabolic adaptations of the mammary gland during lactation. Semi-targeted MRM analysis of metabolites in the mammary gland found 20 increased and 15 decreased in GKO mice (Fig. 7A, B). However, there was no effect of genotype on milk metabolite profiles. Pathway analysis of these metabolites conducted using the Small Molecule Pathway Database (SMPDB), exposed disruptions across multiple metabolic networks (Fig. 7C, D, Supplementary Fig. 1). Notably, several amino acids were elevated among the upregulated metabolites, including the essential amino acids lysine and valine; the conditionally essential amino acids arginine, glycine, and proline; and the non-essential amino acid asparagine. The greater abundance of these amino acids reflects the enrichment in the arginine and proline metabolism pathways. Additionally, the carnitine synthesis pathway was enriched with metabolites more abundant in the mammary gland of GKO mice, including L-carnitine, as recently observed in the liver (32, 33), lysine—an amino acid required in the carnitine biosynthetic pathway—and glycine, a by-product of the carnitine biosynthetic pathway (Fig. 7A–C). The carnitine and acetyl-carnitine also appeared in pathways related to the oxidation of branched, long-chain, and very long-chain fatty acids (Fig. 7A, C), suggesting an increase in fat oxidation in GKO mice.

Functional annotation analysis of genotype-dependent mammary gland metabolites revealed an enrichment of less abundant metabolites within the urea cycle and ammonia recycling pathways, to include key urea cycle reactants aspartic acid, citrulline, and glutamine, alongside a concomitant decrease in uric acid levels (Fig. 7D, Supplementary Fig. 1). The decrease in the levels of aspartic acid, glutamine, and urea also led to enrichment of the purine and pyrimidine metabolism pathways. Similarly, the pentose phosphate pathway was enriched with metabolites lower in abundance in the mammary glands of GKO mice, such as D-Ribulose 5-phosphate and ADP. A decrease in dihydroxyacetone phosphate, a precursor for the glycerol backbone needed for triglyceride synthesis in the mammary gland (34), reflected the enrichment of glycerol phosphate shuttle and glycerolipid metabolism pathways in GKO mice. There was also a decrease in pathways related to phospholipid and lactose synthesis. Together, the metabolomic analysis indicates a reduction in mammary gland biosynthetic pathways and an increase in lipid oxidative pathways, which likely contributed to the decrease in glycerolipid synthesis in the mammary gland of *Scd1*-deficient mice.

## Discussion

Global knockout of the *Scd1* gene, which plays a pivotal role in lipogenesis, profoundly impacted the lactation competence and mammary gland remodeling, marked by diminished mammary gland weight and acini area. Lipidomic analysis revealed that *Scd1* deficiency altered the mammary gland lipidome and also led to diminished milk glycerolipids. The metabolomic analysis supports a decrease in lipid biosynthetic pathways coupled with an increase in oxidative pathways, which may have played a role in the decrease in milk glycerolipids. These alterations likely led to lower neonatal weights. Collectively, our findings provide new insights into the metabolic role of SCD1, highlighting its critical role in regulating mammary gland function and lactation.

Analysis of the lipidomic landscape of the mammary gland revealed alterations due to *Scd1*'s absence. Our findings showed that *Scd1* deficiency altered the profiles of individual species within the TG, DG, and PC classes in the mammary gland. In all classes, alterations in profiles reflected a lower abundance of MUFA chains. Additionally, the carbon chains of the GKO mice were longer, which reflects a greater dependence on fatty acids of dietary origin (31) as well as increased elongation of these fatty acids, which was supported by an upregulation in the expression of mammary gland *Elovl6*.

Analysis of milk revealed a decrease in total milk TG and DG content of GKO mice, as well as alterations in the concentration of the individual TG and DG species, in agreement with findings in the liver (12, 32). Similar to the mammary gland, these decreased lipids had at least one MUFA acyl chain. Also, the profiles of carbon lengths of the TG and DG decreased in the milk of GKO mice were shorter in length. Similar to findings in the mammary gland, these results support the notion that *Scd1* deficiency led to a lower source of de novo synthesized fats and greater dependence on dietary and stored fats to provide milk lipids.

To promote net mammary lipogenesis and ensure adequate lipid availability in the milk, the mammary gland upregulates lipid synthesis while simultaneously downregulating lipid catabolism (8). Metabolomic analysis in this study suggests that *Scd1* deficiency reduces several biosynthetic pathways, including glycerolipid, lactose, and phospholipid synthesis pathways. Conversely, *Scd1* deficiency increased catabolic pathways, notably those related to fatty acid metabolism. Together, the decrease in biosynthetic pathways and the increase in catabolic pathways likely contributed to the reduction in milk glycerolipids under *Scd1* deficiency.

The marked reduction in milk TG and DG of *Scd1-deficient* dams emerges as a pivotal factor contributing to the lower weights in the neonates. During early lactation, the mammary gland assumes a critical role in synthesizing milk rich in TG to provide nutrients for the growth and survival of the pups. Our study revealed a decrease in both mammary gland weight and the area of acini, usually associated with the onset of involution in lactating GKO dams (35). These decreases are likely linked with functionality and architecture defects of the mammary gland, reducing efficient milk lipid synthesis and storage. Milk lipids are a critical component, providing both nutritional support and signaling functions (36). The alterations in the milk profiles of the GKO mice likely reduced the nutritional availability, resulting in lower neonatal weights. Limitations in milk sample size precluded analysis of protein and lactose content. The reduced growth in pups may also be related to changes in these components. Together, these findings uncover a novel role of SCD1 in mammary gland function in shaping the lipid composition of the mammary gland, subsequently impacting milk lipid composition and neonatal development.

Our findings of the reduction of glycerolipids in milk under *Scd1* deficiency are consistent with our previous findings in other tissues, such as the liver, where decreased TG synthesis due to reduced lipogenesis and increased fatty acid oxidation led to lower body adiposity (12, 17, 28, 32). These findings have potential implications in medicine and the agricultural industry. For instance, human SCD polymorphisms influence diabetes and obesity outcomes (https://www.researchsquare.com/article/rs-2657293/v1) (37). Similarly, it is plausible that these polymorphisms could influence mammary gland function during lactation and, subsequently, infant health, particularly during the first six months of early development when exclusive breastfeeding is recommended (38, 39). Furthermore, just as SCD polymorphisms in ruminants affect the fat content in meat (40, 41), it is conceivable that they also influence ruminant milk quality for human and calf consumption.

Our investigations sought to determine the role of SCD1 in the mammary gland during lactation. We found out that *Scd1* deficiency impacted mammary gland morphology, resulting in diminished mammary gland weight and reduced acini area. Moreover, *Scd1* deficiency altered the profile of the mammary gland lipidome and metabolites. *Scd1* deficiency ultimately decreased milk glycerolipid production, which plausibly contributed to the lower weights observed in suckling neonates. Our results underscore the critical importance of SCD1 in orchestrating mammary gland metabolism and lactation, with consequential effects on neonatal growth and development.

## Data availability

All data are contained within the article and the accompanying supplemental data. MRM raw data files were uploaded to PURR (DOI: 10.4231/JA2N-DJ86).

## Supplemental data

This article contains supplemental data.

## Conflict of interest

The authors declare that they do not have any conflicts of interest with the content of this article.
