# Erratum to ‘Stearoyl-CoA desaturase-1 is vital for milk lipid synthesis: Deletion impairs mammary gland and neonatal development’ [Journal of Lipid Research 66/12 (2025) 100941]

**DOI:** 10.1016/j.jlr.2026.101073

**Published:** 2026-07-04

**Authors:** Mugagga Kalyesubula, Jysiane Cardot, Hailey Huff, Daniel Bergman, Kaitlyn O'Donoghue, Veronica Pegkou Christofi, Kaela Groppel, Lucas M. O'Neill, Lucas Lefers, Jacqueline Rose Miller, Ethan Anderson, Madelaine M. Becker, Dylan Cootway, Joshua Walter, Leriana Garcia Reis, Linda Beckett, Christina R. Ferreira, Catherine Mounier, Isabelle Plante, Theresa M. Casey, James M. Ntambi

**Affiliations:** 1 Department of Biochemistry, University of Wisconsin-Madison, Madison, WI, USA; 2 Institut National de la Recherche Scientifique (INRS), Centre Armand-Frappier Santé Biotechnologie, Laval, Québec, Canada; 3 Department of Animal Sciences, Purdue University, West Lafayette, Indiana 47907, USA; 4 Bindley Bioscience Center, Purdue University, West Lafayette, IN, USA; 5 Laboratoire du métabolisme des lipides, CERMO-FC, Département des sciences biologiques, Université du Québec à Montréal, Montréal, Québec H3C 3P8, Canada; 6 Department of Nutritional Sciences, University of Wisconsin-Madison, Madison, WI, USA

The publisher regret that some affiliations in the original publication were incorrect. The affiliations for authors Reis, Beckett, Ferreira, Mounier, Plante, and Casey currently read:

Leriana Garcia Reis^2^, Linda Beckett^2^, Christina R. Ferreira^3^, Catherine Mounier^4^, Isabelle Plante^5^, Theresa M. Casey^2^

They should read:

Leriana Garcia Reis^3^, Linda Beckett^3^, Christina R. Ferreira^4^, Catherine Mounier^5^, Isabelle Plante^2^, Theresa M. Casey^3^

Affiliation 3 currently reads:

Department of Animal Sciences, Purdue University, West Lafayette, IN, USA

It should read:

Department of Animal Sciences, Purdue University, West Lafayette, Indiana 47907, USA

Affiliation 5 currently reads:

Laboratoire du métabolisme des lipides, CERMO-FC, Département des sciences biologiques, Université du Québec à Montréal, Montréal, Québec, Canada

It should read:

Laboratoire du métabolisme des lipides, CERMO-FC, Département des sciences biologiques, Université du Québec à Montréal, Montréal, Québec H3C 3P8, Canada

