# Supplementary material for: Stearoyl-CoA desaturase-1 is vital for milk lipid synthesis: Deletion impairs mammary gland and neonatal development
Source: J Lipid Res. 2025 Nov 11;66(12):100941. doi: 10.1016/j.jlr.2025.100941 (PMC12753282; doi:10.1016/j.jlr.2025.100941)
Supplement: Supplemental Figure 1 [file mmc1.docx]

**
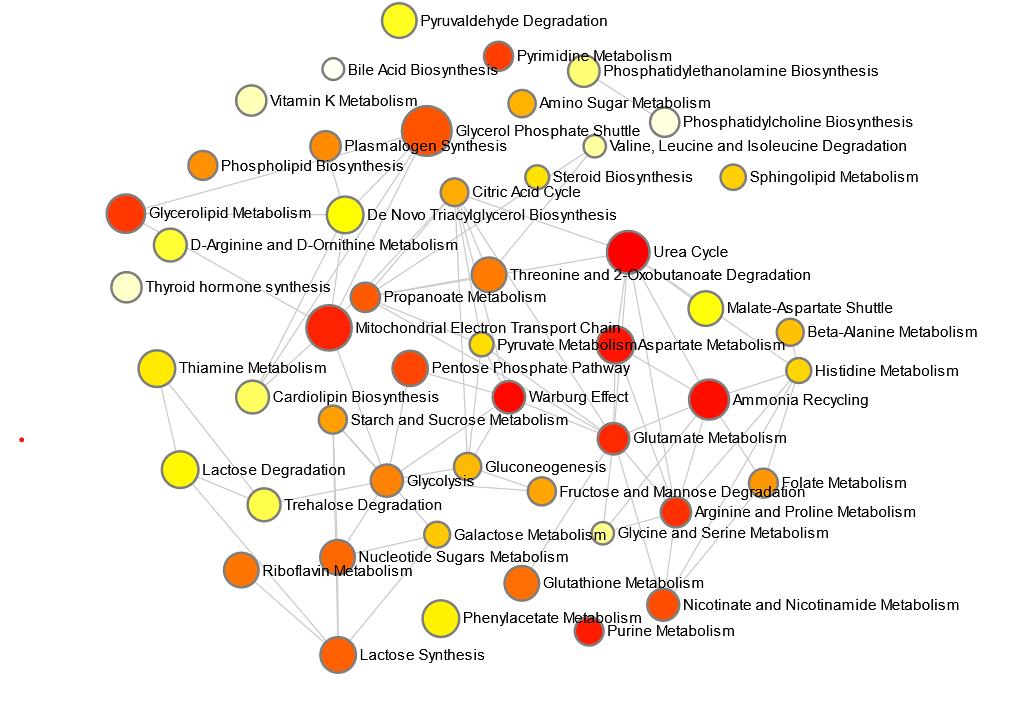
**

**Supplementary Fig 1**: Network analysis of decreased metabolic pathways in *Scd1* global Knockout Mice.
