# Supplementary material for: Stearoyl-CoA desaturase-1 is vital for milk lipid synthesis: Deletion impairs mammary gland and neonatal development
Source: J Lipid Res. 2025 Nov 11;66(12):100941. doi: 10.1016/j.jlr.2025.100941 (PMC12753282; doi:10.1016/j.jlr.2025.100941)
Supplement: Supplemental Table 1 [file mmc2.docx]

**Table S1: Primers**

| **Gene name** | **Forward** | **Reverse** |
| --- | --- | --- |
| *Rps3* | CGG TGC AGA TTT CCA AGA AG | GGA CTT CAA CTC CAG AGT AGC C |
| *Scd1* | TTC TTG CGA TAC ACT CTG GTG C | CGG GAT TGA ATG TTC TTG TCG T |
| *Fas* | GCT GCG GAA ACT TCA GGA AAT | AGA GAC GTG TCA CTC CTG GAC TT |
| *Acc* | TGA CAG ACT GAT CGC AGA GAA AG | TGG AGA GCC CCA CAC ACA |
| *Elovl6* | GAA CAA GCG AGC CAA GTT TG | TGT AAG CAC CAG TTC GAA GAG |
| *Scd2* | CCA CTT GAA AGT AGC CTT AC | ATA GAA TAG GGC CAC AGC TCA |
| *Gpat1* | CAA CAC CAT CCC CGA CAT C | GTG ACC TTC GAT TAT GCG ACT A |
| *Dgat1* | GGC CTT ACT GGT TGA GTC TAT C | GTT GAC ATC CCG GTA GGA ATA A |
